# Residual nitrite dynamics, bioactive responses, and lipid oxidation in beef sausages formulated with fermented radish and beetroot powders during refrigerated storage

**DOI:** 10.1016/j.fochx.2026.104363

**Published:** 2026-08-27

**Authors:** Samar A. Almohaimeed, Fahad Y. AL Juhaimi, Elfadil E. Babiker, Isam A. Mohamed Ahmed

**Affiliations:** a Department of Food Science and Human Nutrition, College of Agriculture and Food, Qassim University, Buraydah 51452, Saudi Arabia; b Department of Food Science and Nutrition, College of Food and Agricultural Sciences, King Saud University, Riyadh 11451, Saudi Arabia

**Keywords:** Natural curing, Pre-converted vegetable, Fermented radish, Fermented beetroot, Residual nitrite, Lipid oxidation, Beef sausage

## Abstract

This study investigated fermented radish and beetroot powders as whole pre-converted vegetable matrices in beef sausages. Six formulations were prepared: a nitrite-free control, a sodium nitrite control (156 mg/kg), and sausages containing 3% or 6% fermented radish/beetroot powder. Samples were analyzed at day 0, day 5, and day 10 for residual nitrite, phenolic content, DPPH activity, TBARS, pH, color, microbial load, and sensory attributes. Sodium nitrite maintained the highest residual nitrite and cured-color response. Among vegetable treatments, 6% fermented radish (R6) showed the highest residual nitrite and phenolic content at day 10, whereas 3% fermented radish (R3) showed the highest DPPH activity and lowest TBARS at day 5. All treated sausages had lower microbial counts than the nitrite-free control at day 5. At day 10, 3% fermented beetroot (B3) showed the lowest TBARS and microbial load, while 6% fermented beetroot (B6) maintained acceptability comparable to sodium nitrite. Overall, fermented radish and beetroot powders showed potential as natural curing matrices for beef sausages.

## Highlights

• Fermented vegetables acted as whole pre-converted curing matrices.
• Radish showed stronger nitrite and bioactive responses.
• R3 gave the best day 5 oxidative response among natural treatments.
• B6 improved late-storage sensory acceptability.
• Responses depended on plant matrix, concentration, and storage time.

## 1 Introduction

Consumer demand for processed meat products formulated with fewer synthetic additives has increased the need for research on natural curing strategies that can maintain the technological and sensory quality attributes in cured meat products (Kim et al., 2025; Melios et al., 2024; Yong et al., 2021). In cured sausages, sodium nitrite remains difficult to replace because its function is not limited to color formation. Nitrite-derived nitric oxide participates in nitrosylated heme pigment formation, supports characteristic cured flavor, slows lipid oxidation, and contributes to the control of dangerous foodborne pathogens, including *Clostridium botulinum* (Yong et al., 2021; Zhang et al., 2023). The reduction or replacement of synthetic nitrite is a challenge because alternative ingredients must support several quality functions within the meat matrix (Kim et al., 2025; Schopfer et al., 2022).

Vegetable-based curing commonly uses nitrate-rich plant materials with nitrate-reducing starter cultures. The conversion step may occur inside the meat batter or before formulation through a pre-converted vegetable ingredient (Schopfer et al., 2022; Yong et al., 2021). This method depends on microbial reduction of nitrate to nitrite and subsequent nitric oxide formation, which then contributes to cured pigment development in the meat system (Schopfer et al., 2022). However, the response is not uniform across meat products. Studies in sausage and emulsion-type meat products show that the efficacy of vegetable-based curing can be affected by plant source, conversion conditions, ingredient level, and product matrix (Hwang et al., 2018; Kim et al., 2025; Ozaki et al., 2021). Therefore, each vegetable matrix should be evaluated in the product where it is expected to be used, as it may not mimic the technological effects of sodium nitrite consistently across different meat systems (Kim et al., 2025; Schopfer et al., 2022).

Radish and beetroot are promising vegetables for natural curing because they can provide nitrate and bioactive compounds that may contribute to curing reactions and quality stability. Ozaki et al. (2021) reported high nitrate contents in radish and beetroot powders, approximately 16,000 and 14,000 mg/kg, respectively. Radish has been reported as a strong nitrate source and has shown curing potential in cooked and fermented meat systems (Bae et al., 2020; Ozaki et al., 2021). Beetroot has also been evaluated in fermented beef sausage because it provides nitrate, phenolic compounds, and betalain pigments, although its strong pigment contribution may influence the visual appearance of cured meat products (Schopfer et al., 2022; Sucu & Turp, 2018). These differences make radish and beetroot useful for comparing how plant matrix composition affects natural curing performance.

Earlier pre-converted nitrite studies in sausage and meat-emulsion systems mainly examined filtered extracts, leaving limited information on whole, unfiltered pre-converted preparations in raw beef sausage (Choi et al., 2017; Hwang et al., 2018; Yong et al., 2021). Further research is needed to determine whether whole fermented radish and beetroot powders can support the quality attributes of beef sausages compared with nitrite-free and conventional- cured formulations during refrigerated storage. This is important because the whole fermented matrix carries not only pre-converted nitrite, but also plant solids and bioactive compounds. The present study evaluated the effects of fermented radish and beetroot powders at matched 3% and 6% levels on physicochemical properties, residual nitrite, color, lipid oxidation, phenolic content, DPPH radical scavenging activity, texture, total viable count, and sensory attributes of beef sausages stored at 4 ± 1 °C for 10 days. The formulations comprised a nitrite-free negative control (NC), a positive control containing 156 mg sodium nitrite/kg (PC), 3% fermented radish (R3), 6% fermented radish (R6), 3% fermented beetroot (B3), and 6% fermented beetroot (B6). Day 5 was used as a post-recommended practical comparison point for raw sausage, and day 10 represented extended storage.

## 2 Materials and methods

### 2.1 Materials

Boneless round beef meat from young male cattle (*Bos taurus*), together with beef back fat from the same carcass, was purchased from a local slaughterhouse in Riyadh, Saudi Arabia, within 1 h postmortem. The meat and fat were transported to the laboratory under chilled conditions. Fresh radishes, beetroots, and spices were obtained from a local market in Riyadh, Saudi Arabia, and transported to the laboratory under the same chilled conditions. All chemicals used in the analyses were of analytical grade.

### 2.2 Samples preparation

#### 2.2.1 Preparation of fermented vegetable powders as pre-converted nitrite sources

Fermented radish and beetroot powders were obtained using the preparation procedure reported by Hwang et al. (2017), with a modification as described previously by Almohaimeed et al. (2026). For each vegetable, 10 g of freeze-dried powder was dispersed separately in 100 mL of double-distilled water for 30 min and inoculated with 0.025% of a mixed *Lactobacillus sakei* and *Staphylococcus carnosus* starter culture (F-RM-52, Bactoferm, Chr. Hansen Inc., Milwaukee, WI, USA). Each suspension was incubated separately with shaking at 30 °C for 24 h. After fermentation, each whole preparation was concentrated separately in a vacuum oven at 60 °C for 21 h to reduce the water carried into the meat formulation while using a relatively low temperature to minimize potential losses of vegetable-derived bioactive compounds. This step enabled the complete fermented preparation, including the vegetable solids, aqueous fraction, and starter culture, to be incorporated into the sausage batter without filtration. The concentrated preparations were stored at 4 °C and added at levels equivalent to 3% or 6% of the initial vegetable powder.

#### 2.2.2 Preparation of beef sausage formulations

Beef sausages were prepared according to the process as described by Choi et al. (2017), with some modifications. Using a Kenwood kitchen mincer (Kenwood Co., Ltd., Havant, UK), the lean and fat beef were minced separately twice for 2 min under chilled conditions. Six beef sausage formulations, each weighing 667 g, were prepared as shown in Table 1. The formulations were NC, PC, R3, R6, B3, and B6. The two controls: negative (NC) and positive (PC) refer to sausages without sodium nitrite and sausages with 156 mg/kg sodium nitrite, respectively. R3 and R6 received the separately fermented radish preparation at 3% and 6%, respectively, whereas B3 and B6 received the separately fermented beetroot preparation at 3% and 6%, with each addition replacing an equivalent portion of water, as shown in Table 1. The PC formulation contained 0.25% of a curing premix composed of 6.25% sodium nitrite and 93.75% NaCl, providing 156 mg sodium nitrite/kg product, consistent with the U.S. maximum ingoing limit for comminuted cured meat products (FSIS, 1995).

**Table 1:** Composition (%) of beef sausage incorporating varying concentrations of fermented radish and beetroot powders.

Ingredients%	Sample groups					
	Control		Beetroot		Radish	
	NC	PC	B3	B6	R3	R6
lean meat	76.70	76.70	76.70	76.70	76.70	76.70
added fat	11	11	11	11	11	11
cold water	10.50	10.25	7.50	4.50	7.50	4.50
salt	1.20	1.20	1.20	1.20	1.20	1.20
Black pepper	0.20	0.20	0.20	0.20	0.20	0.20
Garlic powder	0.20	0.20	0.20	0.20	0.20	0.20
Onion powder	0.20	0.20	0.20	0.20	0.20	0.20
Sodium ascorbate	0.05	0.05	0.05	0.05	0.05	0.05
Curing mixture (6.25% sodium nitrite +93.75% NaCI)	0	0.25	0	0	0	0
Fermented beetroot powder	0	0	3	6	0	0
Fermented radish powder	0	0	0	0	3	6

First, the lean and fat were combined with the spices until a uniform meat batter was obtained. Then the batter was portioned into six equal parts, each part combined with its specific treatment components. The sausage batters were homogenized by a Stephan UM 12 mixer (Stephan U. Sohner GmbH & Co., Hameln, Germany) while maintaining the batter temperature below 10 °C. The batters were then stuffed into 18 mm cellulose casings with sausage-filling equipment.

For sensory evaluation, the sausages were cooked by steaming at 80 °C until the internal temperature was attained 72 °C. Samples were removed immediately upon reaching this temperature. Then, the cellulose casings were removed, and the sausages were heated in an oven for 20 min before serving. For storage stability evaluation, raw sausages from each formulation were packed separately in polyethylene bags and stored at 4 ± 1 °C for up to 10 days. Quality analyses were performed at day 0, day 5, and day 10. Analytical measurements were conducted in triplicate.

The Saudi technical regulation SFDA.FD 150-1/2018 states that the recommended shelf life for chilled uncooked meat and poultry sausages stored in appropriate containers is 3 days under refrigerated conditions. Therefore, day 5 was selected as a practical evaluation point. Day 10 was included as an extended storage condition to test whether treatment effects remained detectable beyond the expected practical storage period.

### 2.3 Determination of nitrate and nitrite by ion chromatography

Nitrate and nitrite concentrations were measured in the radish and the beetroot powders and in their fermented pre-converted vegetable mixtures using ion chromatography. The analysis was based on the validated anion-exchange ion chromatography technique reported by McMullen et al. (2005), with slight modifications in preparation of the samples as described previously by Almohaimeed et al. (2026). In summary, 0.250 g of each sample was extracted with nitrate-free deionised water in a 100 mL volumetric flask, sonicated for 20 min at 37 °C, cooled to ambient laboratory temperature, filtered with a syringe membrane filter, and diluted when required to fit the calibration range.

Chromatographic separation was carried out using a Dionex ICS-6000 system equipped with an IonPac AS19-4 μm RFIC analytical column and IonPac AG19 RFIC guard columns, with a KOH gradient elution program and conductivity detection. Nitrite and nitrate were quantified by external calibration using a certified mixed anion standard, and results were calculated using Chromeleon software and expressed as mg/kg sample.

### 2.4 Determination of residual nitrite

Residual nitrite in sausage samples was quantified using the Griess colorimetric reaction according to Merino (2009), with minor adaptation to the sausage matrix. In this assay, nitrite present in the sample extract was reacted with sulfanilamide for diazotization and then coupled with N-(1-naphthyl) ethylenediamine dihydrochloride to produce a colored azo compound. The absorbance of the developed color was recorded at 540 nm. Sodium nitrite standards were used to construct the calibration curve, and results were reported as mg NO₂^−^/kg sausage after being adjusted according to the dilution.

### 2.5 Chemical composition analysis

Moisture, fat, protein, and ash contents of raw beef sausage samples were determined using AOAC official methods (AOAC, 2007). The corresponding method numbers were 940.09 for moisture, 960.39 for fat, 992.15 for protein, and 942.05 for ash. The proximate composition results were reported as a percentage of raw sample weight.

### 2.6 Determination of phenolic content by HPLC-UV

Phenolic content was quantified by HPLC-UV and reported as gallic acid equivalents. The procedure was adapted from the technique reported by Tapia-Quirós et al. (2025), with slight modifications for the sausage matrix. Briefly, 5.0 g of each sausage sample was mixed with 50 mL of distilled water: acetone (1:1, v/v), and the mixture was extracted in an ultrasonic bath for 35 min at ambient temperature. The extract was then centrifuged at 5000 rpm, and the supernatant was passed through a 0.22 μm syringe filter before HPLC injection. Phenolic content was determined by HPLC at 280 nm using a Thermo Scientific Dionex UltiMate 3000 system with a Nucleodur C18 Gravity column. Separation was performed under isocratic elution with acidified water/acetonitrile, 70:30, and quantification was based on an external gallic acid calibration curve, with results expressed as mg GAE/kg sample.

### 2.7 DPPH radical scavenging activity

The radical-scavenging capacity of the sample extracts was evaluated using the 1,1-diphenyl-2-picrylhydrazyl (DPPH) assay. The procedure was adapted from Singh et al. (2002) and was applied to radish powder, beetroot powder, fermented vegetable mixtures, and beef sausage samples. Briefly, 200 uL of each sample extract was mixed with 800 uL of 0.1 M Tris-HCl buffer (pH 7.4). Then, 1 mL of DPPH solution (250 uM) was added, and the mixture was vortexed. The reaction tubes were kept in the dark at room temperature for 20 min, after which the absorbance was measured at 517 nm. DPPH radical scavenging activity was calculated using the following equation:

$$\text{DPPH radical scavenging activity}\;\left(\%\right)=\left[\left(\text{Ablank}-\text{Asample}\right)/\text{Ablank}\right]\;x\;100$$

### 2.8 Thiobarbituric acid reactive substances (TBARS) analysis

Lipid oxidation was determined using the TBARS assay, according to the technique reported by Rosmini et al. (1996). Briefly, 5.0 g of fresh sausage sample was homogenized with 20 mL of distilled water, sonicated for 10 min, and filtered through Whatman No. 1 filter paper. A 1 mL aliquot of the filtrate was mixed with 4 mL of 20% thiobarbituric acid solution and 100 μL of 10% butylated hydroxytoluene, then placed in a water bath at 100 °C for 10 min for color development. After cooling and centrifugation at 5500 x*g* for 25 min, the supernatant absorbance was measured at 532 nm. Quantification was performed using an external calibration curve prepared from malondialdehyde tetrabutylammonium salt, with correction to free malondialdehyde and its tetrabutylammonium salt using the molecular weight factor 72.06/313.52 = 0.2298. Results were expressed as mg MDA equivalents/kg sample, and analyses were performed in triplicate.

### 2.9 External and internal color measurement

Instrumental color of beef sausage samples was measured using a Hunter Lab colorimeter (Miniscan XE Plus 4500 L, Reston, VA, USA). Color values were recorded according to the CIE system as lightness (L*), redness (a*), and yellowness (b*). Before measurement, the instrument was calibrated with a standard white calibration plate according to the manufacturer's instructions.

For external color, readings were taken directly from the outer surface of the sausage samples. For internal color, each sausage sample was cut in half, and measurements were taken from the freshly exposed internal cross-section. Three readings were obtained from different positions for both the external and internal surfaces of each sample, and the mean value was used for statistical analysis.

### 2.10 Total viable count analysis

Total viable count (TVC) in beef sausage samples during refrigerated storage was determined according to the standard procedure described by the International Commission on Microbiological Specifications for Foods (ICMSF & Clark, 1983), with minor adaptation to the sausage matrix. Briefly, 1 g of the sausage sample was aseptically transferred into 9 mL of sterile 0.1% peptone water and homogenized to obtain the initial dilution. Serial ten-fold dilutions were then prepared by transferring 1 mL of the homogenate into 9 mL of sterile 0.1% peptone water.

Appropriate dilutions were plated using the pour-plate method by adding 1 mL of the selected dilution onto plate count agar. The plates were incubated at 37 °C for 48 h, after which visible colonies were counted. Duplicate plates were prepared for each selected dilution, and the results were expressed as log₁₀ CFU/g sample.

### 2.11 pH measurement

The pH of raw beef sausage samples was measured using a portable meat pH meter equipped with a penetrating electrode (Model HI99163, Hanna Instruments, Woonsocket, RI, USA), following the procedure described by Polizer-Rocha et al. (2020). Before analysis, the pH meter was calibrated with standard buffer solutions at pH 4.01 and 7.01. The electrode was inserted directly into each raw sausage sample at three different positions, and the mean value was used for statistical analysis.

The pH values of radish powder, beetroot powder, and the fermented vegetable mixtures were determined separately by dispersing 1 g of each sample in 10 mL of distilled water. The suspension was then measured using a pH meter probe (Corning Scientific Products, New York, NY, USA).

### 2.12 External and internal texture profile analysis

Texture profile analysis (TPA) of raw beef sausage samples was performed using a Brookfield CT3 texture analyzer (Brookfield Engineering Laboratories, Middleboro, MA, USA), following the principles described by Bourne (1978). Measurements were conducted at each storage interval for both the external and internal of the sausage samples.

For external texture measurement, intact sausage samples were placed horizontally on the analyzer platform, and compression was applied directly to the outer surface while the cellulose casing remained on the sample. For internal texture measurement, each sausage sample was cut in half, positioned vertically with the freshly exposed internal cross-section facing the probe, and compressed from the cut surface. The cellulose casing remained on the lateral side of the sample during internal measurement.

A two-cycle compression test was used to determine hardness (kg), cohesiveness, springiness (mm), and chewiness, calculated as hardness x cohesiveness x springiness. Measurements were performed in triplicate for each treatment and storage time, and the mean value was used for statistical analysis.

### 2.13 Sensory analysis

Sensory evaluation of cooked beef sausages was performed at day 0, day 5, and day 10 of cold storage. The sensory panel consisted of twenty semi-trained panelists (30% female and 70% male, aged 20 to 40 years). The panel members were undergraduate and graduate students of the Department of Food Science and Nutrition, College of Food and Agricultural Sciences, King Saud University who had previous experience in sensory-evaluation of meat products. Screening was therefore based on this previous sensory experience, together with self-declared absence of food allergy, absence of smoking, absence of taste or smell impairment, and willingness to attend all three storage sessions. Before the first session, panelists received a short orientation covering the definition of each attribute assessed in this study and the correct use of the 9-point hedonic scale.

The panelists evaluated the sausage samples using a 9-point hedonic scale, ranging from 1, extremely dislike, to 9, extremely like. The assessed sensory attributes included color, taste, texture, flavor, tenderness, and overall acceptability. Samples were sliced into 1-cm-thick pieces, labeled with random three-digit codes, and served in a randomized order to minimize presentation bias. The sensory evaluations were conducted in the sensory test laboratory at the Department of Food Science and Nutrition, College of Food and Agricultural Sciences, King Saud University, which is equipped with partitioned individual booths and standardized lighting conditions. Panelists were provided with water between samples to cleanse the palate. The same panelists participated in all storage-time evaluations. Individual scores were used for statistical analysis, whereas the tables present the mean panel scores. Mean scores of 5 or higher were considered satisfactory, as described by Al-Juhaimi et al. (2018). Before sensory analysis, all participants provided written informed consent, which covered the purpose of the study, the voluntary nature of participation, and the right to withdraw at any time without giving a reason. The sensory evaluation was conducted with approval from the King Saud University Ethics Committee (IRB Approval of Research Project No. E-25-10195).

### 2.14 Statistical analysis

Statistical analyses were performed using IBM SPSS Statistics for Windows, version 31.0.2.0 (IBM Corp., Armonk, NY, USA). All analytical variables were analyzed separately using a two-way analysis of variance (ANOVA), with treatment, storage day, and their interaction included as fixed effects. When the interaction was significant (*P* < 0.05), simple main effects were examined, followed by Duncan's multiple range test for mean separation at *P* < 0.05. When the interaction was not significant, main effects were compared using Duncan's multiple range test at *P* < 0.05. Results are expressed as mean ± standard deviation (SD). Sensory attributes were analyzed separately using a linear mixed model, with treatment, storage day, and their interaction as fixed effects and panelist as a random effect. Pairwise comparisons of estimated marginal means were adjusted using Sidak's test at *P* < 0.05.

## 3 Results and discussion

### 3.1 Characterization of radish and beetroot powders and their fermented mixtures

The fermented radish and beetroot mixture powders used in the present sausage formulations were previously characterized and published in Almohaimeed et al. (2026). In brief, radish powder was the richer initial nitrate source, whereas fermented beetroot produced a higher nitrite concentration and slightly greater DPPH radical scavenging activity after fermentation. Fermentation also lowered the pH of both vegetable matrices. These results indicate that the fermented mixtures provided pre-converted nitrite and bioactive compounds, with responses depending on the vegetable matrix and not only on initial nitrate concentration. The subsequent sections discuss how these ingredients influenced sausage quality during refrigerated storage.

Table 2 summarizes the characterization data for the vegetable materials. Measured nitrate concentration decreased by 90.8% in beetroot and 93.8% in radish after fermentation. These percentages represent relative decreases in measured nitrate concentration.

**Table 2:** Nitrate, nitrite, pH, and DPPH characteristics of radish and beetroot powders before and after fermentation.

Sample	Nitrate, NO₃^−^ (mg/kg)	Nitrite, NO₂^−^ (mg/kg)	pH	DPPH inhibition (%)	Nitrate decrease (%)
Beetroot powder	3805.42	<LOD	6.56	NA	NA
Fermented beetroot	351.06	751.99	4.40	67.56	90.8
Radish powder	25,623.60	<LOD	6.16	NA	NA
Fermented radish	1588.72	389.11	3.81	63.36	93.8

Values are means of triplicate samples. LOD, limit of detection; NA, not analyzed. Fermentation was performed for 24 h at 30 °C. Nitrate decrease (%) = [(initial nitrate concentration - post-fermentation nitrate concentration)/initial nitrate concentration] × 100. Data source: (Almohaimeed et al., 2026).

### 3.2 Residual nitrite content

Table 3 indicates that treatment, storage time, and their interaction had significant effects on residual nitrite content. As expected, PC showed the highest residual nitrite throughout storage, increasing from 64.21 mg/kg at day 0 to 80.93 mg/kg at day 5 and then decreasing to 22.90 mg/kg at day 10. This clear separation from the natural treatments is consistent with the higher ingoing nitrite in PC, which was 156 mg/kg sodium nitrite. The difference between ingoing and residual nitrite is expected because only part of the added nitrite remains analytically detectable after processing and storage. Sucu and Turp (2018) reported that residual nitrite after production was about 75% of the initial amount and later declined to 4 to 10 mg/kg by the end of storage, while Ozaki et al. (2021) reported that only 62.1% of the ingoing nitrite remained after sausage processing. This reduction has been linked to reactions of nitrite with myoglobin, proteins, and lipids, together with thermal degradation during processing and storage-related changes (Serdaroğlu et al., 2023; Sucu & Turp, 2018).

**Table 3:** Residual nitrite content, phenolic content, DPPH radical scavenging activity, and TBARS values of beef sausages treated with fermented radish and beetroot powders during refrigerated storage.

Parameter	Storage	Treatments						Pₜ	Pₛ	Pₜ × ₛ
		NC	PC	R3	R6	B3	B6			
NO₂^−^ (mg/kg)	0	2.16 ± 0.33^cA^	64.21 ± 0.77^aB^	3.22 ± 0.23^bA^	3.08 ± 0.28^bB^	2.16 ± 0.11^cA^	3.22 ± 0.38^bA^	⁎⁎⁎	⁎⁎⁎	⁎⁎⁎
	5	2.13 ± 0.23^cA^	80.93 ± 0.82^aA^	3.26 ± 0.22^bA^	3.15 ± 0.11^bB^	2.56 ± 0.17^bcA^	3.22 ± 0.13^bA^			
	10	2.27 ± 0.11^cA^	22.90 ± 0.77^aC^	1.95 ± 0.11^cB^	3.69 ± 0.22^bA^	2.20 ± 0.28^cA^	2.24 ± 0.23^cB^			

Phenolic content (mg GAE/kg sample)	0	266.80 ± 1.71^cA^	203.33 ± 4.51^eA^	454.50 ± 4.92^bA^	654.73 ± 8.51^aA^	243.83 ± 5.35^dB^	183.03 ± 5.66^fB^	⁎⁎⁎	⁎⁎⁎	⁎⁎⁎
	5	146.73 ± 3.26^eB^	169.13 ± 3.41^dB^	254.23 ± 4.41^bC^	451.33 ± 4.01^aC^	148.10 ± 6.26^eC^	183.80 ± 5.70^cB^			
	10	108.67 ± 8.50^fC^	123.63 ± 4.97^eC^	420.80 ± 3.27^bB^	540.03 ± 3.93^aB^	256.73 ± 5.85^cA^	216.90 ± 5.76^dA^			

DPPH (% inhibition)	0	56.37 ± 0.22^dB^	60.31 ± 0.38^cB^	24.94 ± 0.22^fC^	74.43 ± 0.38^aA^	69.09 ± 0.01^bB^	30.92 ± 0.38^eB^	⁎⁎⁎	⁎⁎⁎	⁎⁎⁎
	5	34.35 ± 0.38^eC^	44.53 ± 0.22^dC^	75.17 ± 0.05^aA^	55.09 ± 0.22^cC^	58.02 ± 0.38^bC^	55.34 ± 0.38^cA^			
	10	78.25 ± 0.01^bA^	79.39 ± 0.38^aA^	74.31 ± 0.23^dB^	72.01 ± 0.22^eB^	75.95 ± 0.04^cA^	55.09 ± 0.22^fA^			

TBARS (mg MDA equivalents/kg)	0	1.40 ± 0.03^aA^	0.43 ± 0.03^cA^	0.45 ± 0.03^cB^	0.64 ± 0.07^bB^	0.46 ± 0.05^cB^	1.38 ± 0.04^aA^	⁎⁎⁎	⁎⁎⁎	⁎⁎⁎
	5	0.38 ± 0.01^dC^	0.25 ± 0.05^eB^	0.39 ± 0.05^dB^	1.37 ± 0.04^aA^	0.67 ± 0.03^cA^	1.26 ± 0.03^bB^			
	10	0.56 ± 0.03^cB^	0.11 ± 0.04^eC^	0.54 ± 0.03^cA^	0.75 ± 0.05^bB^	0.35 ± 0.06^dC^	1.03 ± 0.03^aC^			

Data is presented as mean ± SD. Within each storage day, different lowercase superscripts indicate significant differences among formulations; within each formulation, different uppercase superscripts indicate significant differences among storage days (*p* < 0.05). NC, negative control; PC, positive control; R3 and R6, fermented radish at 3% and 6%; B3 and B6, fermented beetroot at 3% and 6%. Pₜ, treatment effect; Pₛ, storage effect; Pₜ × ₛ, treatment × storage interaction.

⁎⁎⁎ *P* < 0.001.

Among the natural treatments, residual nitrite remained low and generally close to NC across storage. At day 10, R6 had the highest residual nitrite among the vegetable treatments (3.69 mg/kg), whereas R3, B3, and B6 remained close to NC.

An important point is that residual nitrite in sausage did not directly mirror the nitrite concentration of the fermented powders. Although fermented beetroot showed higher nitrite than fermented radish, beetroot treatments did not consistently produce higher residual nitrite in the sausage, while R6 reached the highest value among the natural treatments at day 10. At the same time, radish powder initially contained much more nitrate than beetroot powder, which may have provided a larger amount of unconverted nitrate still available in the radish-based treatments after pre-conversion. This showed that residual nitrite in the final product was influenced by nitrate-to-nitrite conversion efficiency and by resulting nitrite reactions within the meat matrix. In the present study, this may also be linked to the use of the whole fermented vegetable mixture powder together with the starter culture rather than a filtered extract, so some nitrate retained in the vegetable matrix may have remained available for additional microbial reduction to nitrite within the sausage system. This conclusion agrees with reports that nitrate-containing plants can be applied directly in meat formulations with nitrate-reducing microorganisms and an incubation period (Schopfer et al., 2022; Serdaroğlu et al., 2023). However, this should be considered cautiously, because residual nitrite is also reduced by reactions with myoglobin, proteins, and lipids, as well as by processing and storage-related changes (Serdaroğlu et al., 2023; Sucu & Turp, 2018).

Published Saudi research on pre-converted vegetable-derived nitrite in meat products remains limited. To our knowledge, the closest local work is our previous work, in which fermented radish and beetroot powders were incorporated separately at 3% and 6% into beef burgers (Almohaimeed et al., 2026). Other relevant Saudi evidence is limited to the analytical survey of Siddiqui et al. (2018), who used UPLC-MS to quantify nitrite in processed meat and poultry products marketed in the Riyadh region, without manufacturing meat products. Studies conducted internationally employed different ingredient preparations and meat systems. Choi et al. (2017) evaluated a filtered and concentrated pre-converted red beet extract, with or without ascorbic acid, in a pork meat emulsion. In contrast, Sucu and Turp (2018) added nitrate-rich beetroot powder directly to Turkish dry-fermented beef sausage without prior conversion, while Ozaki et al. (2021) directly incorporated dried radish or beetroot powders into fermented dry sausages, allowing nitrite formation during ripening. The present study differs from these approaches by incorporating separately pre-converted, whole, and unfiltered radish and beetroot matrices into beef sausage. Thus, pre-converted nitrite entered the sausage together with the retained vegetable solids, dietary fiber, bioactive compounds, aqueous fraction, and starter culture. This complete matrix may have contributed to the treatment- and storage-dependent residual nitrite response through continuing interactions among residual nitrate and nitrite, vegetable constituents, and the meat matrix during storage.

Overall, none of the natural treatments approached PC in the residual nitrite level. Therefore, the natural systems did not reproduce the synthetic control in residual nitrite amount, but R6 showed the clearest ability to maintain residual nitrite by day 10 among the vegetable-based treatments. This should still be considered as a parameter-specific advantage only, because lower residual nitrite in natural systems has been reported in some studies and mainly reflects their lower initial nitrite availability and different curing kinetics relative to conventional sodium nitrite systems (Jeong et al., 2020; Sucu & Turp, 2018). N-nitrosamines were not analyzed; therefore, the lower residual nitrite values of the natural treatments cannot be interpreted as evidence of lower nitrosamine formation or improved safety.

### 3.3 Chemical compositions

Table 4 shows that moisture, protein, and ash were significantly affected by treatment, storage time, and their interaction, whereas fat was significantly affected by treatment and treatment x storage interaction, but not by storage time alone. Overall, the fermented vegetable powder changed the proximate composition of beef sausages during refrigerated storage, indicating a treatment-dependent storage response. This behavior is consistent with plant-based reformulation studies showing that plant-based powder modifies the balance between water and solids in meat systems, thereby affecting the relative proportions of moisture, protein, fat, and ash (Hawashin et al., 2016; Ozaki et al., 2021).

**Table 4:** Chemical composition (%) of beef sausages treated with fermented radish and beetroot powders during refrigerated storage.

Parameter	Storage	Treatments						P_t_	P_s_	P_txs_
		NC	PC	R3	R6	B3	B6			
Moisture	0	67.97 ± 0.21^dC^	68.13 ± 0.27^dC^	72.75 ± 0.13^aA^	66.82 ± 0.32^eC^	70.48 ± 0.50^bAB^	68.81 ± 0.47^cA^	⁎⁎	⁎⁎	⁎⁎
	5	71.40 ± 0.62^aB^	70.19 ± 0.46^bB^	70.48 ± 0.20^bB^	68.25 ± 0.42^cB^	69.65 ± 0.63^bB^	67.50 ± 0.50^cB^			
	10	72.58 ± 0.09^aA^	72.92 ± 0.31^aA^	70.80 ± 0.36^bB^	72.70 ± 0.27^aA^	70.71 ± 0.21^bA^	68.86 ± 0.45^cA^			

Protein	0	24.74 ± 0.29^bA^	25.18 ± 0.21^aA^	19.27 ± 0.09^dA^	21.70 ± 0.25^cA^	19.00 ± 0.17^dA^	19.05 ± 0.14^dA^	⁎⁎	⁎⁎	⁎⁎
	5	20.71 ± 0.20^aB^	20.04 ± 0.10^bB^	18.04 ± 0.20^cB^	17.26 ± 0.33^dC^	18.04 ± 0.37^cB^	18.27 ± 0.33^cB^			
	10	18.00 ± 0.45^cC^	17.39 ± 0.23^dC^	17.78 ± 0.41^cdB^	20.16 ± 0.26^aB^	18.66 ± 0.08^bA^	17.45 ± 0.31^cdC^			

Fat	0	3.30 ± 0.12^cdB^	3.94 ± 0.31^bA^	3.66 ± 0.08^bcB^	4.01 ± 0.07^bA^	3.18 ± 0.35^dB^	4.53 ± 0.14^aA^	⁎	ns	⁎⁎
	5	4.73 ± 0.21^aA^	2.74 ± 0.09^eB^	3.99 ± 0.11^bA^	3.59 ± 0.13^cB^	3.12 ± 0.10^dB^	3.30 ± 0.23^dB^			
	10	3.69 ± 0.27^bcB^	4.03 ± 0.06^abA^	3.39 ± 0.11^cC^	3.84 ± 0.05^abcA^	4.43 ± 0.48^aA^	2.74 ± 0.59^dB^			

Ash	0	1.50 ± 0.06^aA^	1.34 ± 0.02^bA^	1.58 ± 0.05^aA^	1.00 ± 0.04^cB^	1.38 ± 0.06^bA^	0.95 ± 0.03^cB^	⁎⁎	⁎⁎	⁎⁎
	5	1.00 ± 0.04^cB^	1.19 ± 0.01^abB^	1.21 ± 0.05^aB^	1.11 ± 0.01^bA^	0.90 ± 0.07^dC^	1.20 ± 0.06^aA^			
	10	1.06 ± 0.02^dB^	1.28 ± 0.07^bA^	1.55 ± 0.06^aA^	0.93 ± 0.04^eC^	1.21 ± 0.05^bcB^	1.12 ± 0.06^cdA^			

Data is presented as mean ± SD. Within each storage day, different lowercase superscripts indicate significant differences among formulations; within each formulation, different uppercase superscripts indicate significant differences among storage days (*p* < 0.05). NC, negative control; PC, positive control; R3 and R6, fermented radish at 3% and 6%; B3 and B6, fermented beetroot at 3% and 6%, Pt; treatment, Ps; storage, Ptxs; treatment vs storage. NS-not significant.

⁎ P < 0.05.

⁎⁎ *P* < 0.001.

The lower moisture generally observed in the 6% treatments is consistent with a water replacement effect, because the fermented vegetable powder replaced part of the added water while increasing total solids in the formulation. Previous study in beef patties showed that increasing plant-based powder concentration reduced moisture because of the rise in total solids (Hawashin et al., 2016). Likewise, Ozaki et al. (2021) reported that the addition of radish or beetroot affected moisture content, weight loss, and water activity in fermented sausages, which was related to the fiber-rich vegetable matrix. Therefore, the moisture pattern in the present study likely reflects the combined effects of partial water replacement and higher plant solids.

Protein content was significantly affected by treatment, storage time, and their interaction. At day 0, PC and NC showed the highest protein percentages, at 25.18% and 24.74%, respectively, whereas lower values were recorded in the vegetable-treated sausages, particularly B3 (19.00%), B6 (19.05%), and R3 (19.27%), with R6 showing an intermediate value (21.70%). Because the fermented vegetable powder replaced part of the added water, the initially higher protein percentage in PC and NC is explained as a wet-basis compositional effect. This explanation is supported by the moisture data, where NC and PC had relatively lower moisture contents at day 0 (67.97% and 68.13%), together with higher protein, whereas R3 and B3 had higher moisture contents (72.75% and 70.48%), together with lower protein. Similar compositional shifts associated with plant-based powder incorporation and changes in moisture content in meat systems were discussed by Hawashin et al. (2016). In addition, Kim et al. (2025) noted that plant-derived ingredients such as dietary fibers can modify water-holding capacity in processed meat systems. Therefore, the lower protein values in the natural treatments are more probably explained as the result of both moisture-related dilution and compositional redistribution caused by the incorporation of fermented vegetable powder. The particularly low protein values in B3 and B6 at day 0 may also reflect compositional differences between radish and beetroot powders. Ozaki et al. (2021) reported that radish powder had higher ash and protein contents than beetroot powder, while beetroot powder showed a relatively higher carbohydrate content. This agrees with the present ash data, where beetroot treatments showed lower ash than the radish treatments at the same addition level, 1.38% in B3 versus 1.58% in R3 and 0.95% in B6 versus 1.00% in R6 at day 0.

Fat content was affected by treatment and the treatment × storage interaction, whereas storage alone was not significant (Table 4); the individual values are therefore not repeated in the text.

Ash content was significantly affected by treatment, storage time, and their interaction. R6 showed lower ash values, particularly at day 10 (0.93%), despite the higher inclusion level. This showed that ash content did not increase linearly with fermented vegetable powder level and may have been affected by formulation differences, moisture changes, and matrix distribution during storage. By day 10, R3 remained higher than both NC (1.06%) and PC (1.28%), indicating that the 3% radish treatment contributed more consistently to mineral-related composition than the other natural treatments.

Overall, the proximate composition results show that fermented radish and beetroot powders modified the sausage composition in a treatment-dependent manner. R6 showed the highest protein content at day 10 (20.16%). Fat values fluctuated across treatments and were not directly aligned with TBARS, confirming that lipid oxidation should be interpreted together with matrix structure, residual nitrite, plant-derived compounds, and storage time.

### 3.4 Phenolic content and DPPH radical scavenging activity

Table 3 shows that the phenolic content and DPPH radical scavenging activity were significantly affected by treatment, storage time, and their interaction, indicating a dependent storage response. The radish-based treatments showed the strongest phenolic enrichment throughout storage, with R6 consistently recording the highest values, followed by R3. The greater phenolic content in the radish treatments suggests that the fermented radish powder contributed more extractable phenolic-related material than the fermented beetroot powder under the present processing conditions. This agrees with Guimarães et al. (2021), who reported that Japanese radish curing materials contained phenolic compounds and showed antioxidant capacity. In radish and beetroot powders used for fermented dry sausage, Ozaki et al. (2021) reported slightly higher total phenolic content in radish powder than in beetroot powder, 301.11 versus 292.96 mg GAE/100 g, respectively.

The storage pattern of the phenolic content also suggests that the measured values were influenced not only by the amount introduced with the fermented vegetable powder, but also by storage-related changes in extractability. NC and PC declined progressively from 266.80 to 108.67 mg GAE/kg and from 203.33 to 123.63 mg GAE/kg, respectively. In contrast, R3 and R6 decreased at day 5 and then increased again by day 10, and a similar late increase was also observed in B3 and B6. This response may be related to the use of the fermented vegetable powders as a whole mixture rather than filtered extracts, meaning that plant solids, residual moisture, pigments, phenolic-related compounds, and microbial fermentation products were incorporated into the sausage batter together. In muscle foods, polyphenols can interact with heme proteins through hydrogen bonding and hydrophobic interactions, which may affect protein stability and heme accessibility (Wu et al., 2024). Phenolic compounds can interact with proteins through reversible non-covalent associations and, following phenolic oxidation, through irreversible covalent bonding. These interactions are influenced by factors including pH, salt concentration, protein and phenolic structure and may affect the analytically measured phenolic fraction (Ozdal et al., 2013). Therefore, the increases observed at day 10 in R3, R6, B3, and B6 may reflect storage-related changes in phenolic extractability or protein–phenolic associations. Because free and protein-bound phenolic fractions were not measured separately, this interpretation remains a hypothesis.

DPPH radical scavenging activity showed a significant treatment-dependent storage response, but its ranking did not directly parallel the phenolic content (Table 3).

The phenolic content and DPPH activity did not follow the same pattern. At day 5, R6 showed the highest phenolic content (451.33 mg GAE/kg), but its DPPH inhibition was only 55.09%, whereas R3 showed a lower phenolic content (254.23 mg GAE/kg) but the highest DPPH inhibition (75.17%). A comparable pattern was also observed in the beetroot treatments, where B6 showed a higher phenolic content than B3 at day 5 (183.80 versus 148.10 mg GAE/kg), but DPPH inhibition was slightly lower in B6 than in B3 (55.34% versus 58.02%). These results suggest that DPPH inhibition in sausage was influenced by the overall extractable reducing fraction, not phenolic content alone. This agrees with evidence from meat systems showing that antioxidant capacity assays can be affected by extraction conditions, assay principle, processing, product formulation, and storage-related changes (Echegaray et al., 2021; Umaraw et al., 2024). In nitrite-reduced chicken sausages, Özer et al. (2025) reported that pomegranate flower extract produced a larger increase in total phenolic content than in antioxidant activity, suggesting that higher phenolic content may not translate proportionally into DPPH response in meat systems. Thus, the present pattern may reflect a matrix-dependent response involving phenolics together with other extractable reducing compounds and plant-derived pigments, whose measured activity can vary with concentration, extraction conditions, and interactions within the sausage matrix.

The divergence between phenolic content and DPPH inhibition is analytically plausible because the two measurements describe different properties. Under the applied HPLC-UV procedure, the reported phenolic content reflects the water-acetone-extractable response detected at 280 nm and quantified against an external gallic acid calibration. The method did not identify individual phenolic compounds and should not be interpreted as quantifying all phenolic compounds present in the sausage. In contrast, the DPPH assay measures the ability of compounds present in the assay extract to reduce the stable synthetic DPPH radical. Its response is affected by extraction conditions, reaction kinetics, steric accessibility, and extract composition, with electron-transfer reactions generally predominating under commonly applied conditions (Echegaray et al., 2021).

The increase in DPPH during storage, particularly in NC and PC, suggests that the assay may have been influenced by storage-related changes in extractable reducing compounds, since DPPH reflects radical-scavenging capacity in the extracted medium and does not necessarily indicate lipid oxidative stability within the meat matrix (Echegaray et al., 2021). This interpretation is relevant because NC and PC did not contain fermented vegetable powders, but both treatments contained the common base formulation, including sodium ascorbate and spices; sodium ascorbate is a reducing agent commonly used in cured meat systems to support nitrite reduction to nitric oxide and oxidative stability (Kim et al., 2025). In NC, DPPH inhibition increased from 56.37% at day 0 to 78.25% at day 10, while phenolic content declined from 266.80 to 108.67 mg GAE/kg. A similar late increase was observed in PC, where DPPH inhibition increased from 60.31% at day 0 to 79.39% at day 10, despite the decline in phenolic content from 203.33 to 123.63 mg GAE/kg and the reduction in residual nitrite from 80.93 mg/kg at day 5 to 22.90 mg/kg at day 10. Therefore, the late increase in DPPH in these treatments may reflect the presence or improved extractability of non-phenolic reducing compounds, including residual sodium ascorbate, reducing compounds from the spice formulation, and meat-derived low-molecular-weight compounds released or made more extractable during storage. Meat proteins can generate peptides with radical-scavenging activity after proteolysis, and such peptides may contribute through hydrogen atom donation, electron transfer, and metal-chelating mechanisms (Liu et al., 2016). In PC, nitrite-related reactions may also have contributed to changes in the extractable reducing environment, since nitrite is highly reactive in meat systems and can be converted into nitric oxide and other nitrogen oxide species during curing reactions (Zhang et al., 2023). Accordingly, DPPH inhibition in the present study should be interpreted as the reducing capacity of the assay extract rather than as a direct measure of lipid oxidative stability within the intact sausage matrix. The DPPH radical differs from the radicals involved in lipid peroxidation, and the assay response may be affected by extraction conditions and spectrophotometric interference in complex, colored food matrices (Echegaray et al., 2021). This distinction helps explain why phenolic content and DPPH inhibition did not show the same treatment ranking, as they represent distinct analytical responses rather than interchangeable measures of antioxidant behavior. Therefore, the DPPH results should be interpreted alongside the TBARS results.

Overall, the bioactive results indicate that the fermented radish powder, particularly R6, was more effective in enriching the sausage matrix with phenolic content, whereas DPPH activity did not directly follow phenolic content. At day 5, R6 showed the highest phenolic content (451.33 mg GAE/kg), while R3 showed the highest DPPH inhibition (75.17%). These indicate that phenolic content and DPPH inhibition describe different aspects of the extractable reducing fraction. However, their practical relevance should be interpreted together with lipid oxidation and other quality indicators, which are discussed in the following sections.

### 3.5 Lipid oxidation, TBARS

Table 3 shows that TBARS values were significantly affected by treatment, storage time, and their interaction, indicating that lipid oxidation was dependent on both formulation and storage stage. PC showed the lowest TBARS values throughout storage, decreasing from 0.43 mg MDA equivalents/kg at day 0 to 0.25 mg/kg at day 5 and 0.11 mg/kg at day 10. This pattern indicates that the sodium nitrite treatment provided the strongest inhibition of lipid oxidation under the present sausage conditions. The antioxidant role of nitrite in cured meat systems is mainly related to nitrite-derived nitric oxide, which can interact with heme iron and reduce iron-mediated lipid oxidation in the meat matrix (Zhang et al., 2023). In contrast, NC showed the highest TBARS value at day 0 (1.40 mg/kg), which suggests greater initial exposure to oxidation in the absence of added nitrite or fermented vegetable matrix. However, the later decrease in measured TBARS in NC at day 5 (0.38 mg/kg), followed by a moderate value at day 10 (0.56 mg/kg), should not be explained as a reversal of oxidation. Rather, TBARS reflects MDA-reactive substances, and MDA may further react with proteins, amino groups, and other meat components during storage, which can influence the amount detected by the assay (Chen et al., 2023; Papastergiadis et al., 2012).

Among the natural treatments, lipid oxidation varied significantly according to vegetable source, addition level, and storage time. At day 0, R3 and B3 showed low TBARS values comparable to PC, at 0.45 and 0.46 mg/kg, respectively. By day 10, B3 showed the lowest TBARS among the natural treatments (0.35 mg/kg), and R3 (0.54 mg/kg) showed a lower value than R6 (0.75 mg/kg) and B6 (1.03 mg/kg). Therefore, increasing the fermented vegetable powder from 3% to 6% did not necessarily improve oxidative stability. Instead, the 3% level, especially R3 at day 5 and B3 at day 10, appeared to provide a better lipid oxidation response than the corresponding 6% treatments.

The TBARS response was not directly explained by the proximate fat content. For example, at day 5, NC had the highest fat content (4.73%) but low TBARS (0.38 mg MDA/kg), whereas R6 and B6 showed lower fat contents (3.59% and 3.30%) but higher TBARS values (1.37 and 1.26 mg MDA/kg, respectively). Similarly, at day 10, B3 had the highest fat content (4.43%) but the lowest TBARS among the natural treatments (0.35 mg MDA/kg). This interpretation agrees with Wang et al. (2021), who measured lipid content and TBARS in ground beef patties and found that lipid content was not significantly correlated with initial TBARS, although it became moderately associated with TBARS after display storage. Mechanistically, lipid oxidation in muscle foods is strongly influenced by heme proteins such as myoglobin and hemoglobin, iron-mediated reactions, antioxidant distribution, and interactions within the meat matrix (Wu et al., 2024). Therefore, the present fat values should be interpreted as proximate composition changes, while TBARS should be discussed as a matrix-dependent lipid oxidation response.

The higher TBARS values observed in R6 and B6 indicate that increasing the fermented vegetable powder from 3% to 6% did not produce a linear improvement in lipid oxidation control. At day 5, R3 remained low in TBARS (0.39 mg MDA/kg), whereas R6 increased to 1.37 mg MDA/kg. A similar pattern was observed in the beetroot treatments, where B3 showed 0.67 mg MDA/kg at day 5 and 0.35 mg MDA/kg at day 10, while B6 showed higher values at both points, 1.26 and 1.03 mg MDA/kg, respectively. These results suggest that the 3% level provided a better oxidative response than the 6% level. This pattern was not attributable simply to fat content, because the 6% treatments contained less fat than NC at day 5 but showed greater lipid oxidation. This non-linear response is consistent with previous reports showing that the amount of plant-derived antioxidant added to meat products is a critical factor and that higher levels do not always produce greater inhibition of lipid oxidation. Ferysiuk and Wójciak (2020) noted that higher quantities of natural antioxidants may not always reduce MDA formation in meat systems, and that some combinations of plant extracts with nitrite can show antagonistic responses in TBARS. Jayawardana et al. (2011) reported that 0.2% adzuki bean extract was the optimal level for inhibiting lipid oxidation in pork sausage, although higher levels of 0.3, 0.4, and 0.5% were also tested. This supports the present interpretation that a lower or moderate inclusion level may provide a better oxidative response than a higher level, depending on the meat matrix and antioxidant system. This may be related to how antioxidant compounds are distributed and interact within the sausage matrix. In muscle foods, lipid oxidation is strongly affected by heme proteins, particularly myoglobin and hemoglobin, and by iron-mediated oxidation pathways (Wu et al., 2024). Therefore, antioxidant compounds that contribute to phenolic content or DPPH activity in an extracted medium may not necessarily be located effectively in the lipid phase or close enough to the heme-driven oxidation sites to suppress TBARS formation. This helps explain why R6, despite having the highest phenolic content at day 5 (451.33 mg GAE/kg), also showed the highest TBARS value at the same time (1.37 mg MDA/kg). The same interpretation applies to B6, which did not show better TBARS control than B3 despite the higher inclusion level. Thus, the elevated TBARS values in R6 and B6 are better discussed as matrix-dependent effects of the 6% whole fermented vegetable systems.

Residual nitrite alone did not explain lipid oxidation in the natural treatments. At day 5, residual nitrite values among vegetable-treated sausages were not different statistically, whereas TBARS differed markedly: R3 remained low (0.39 mg MDA/kg), while R6 reached 1.37 mg MDA/kg; similarly, B3 was lower than B6 (0.67 versus 1.26 mg MDA/kg). This showed that lipid oxidation was more influenced by the concentration and behavior of the whole fermented vegetable matrix. This interpretation agrees with reports showing that vegetable-based nitrite alternatives can affect the meat matrix beyond nitrite formation, including moisture, water activity, color, and lipid oxidation (Ozaki et al., 2021), and that higher levels of plant-derived antioxidants do not always result in lower MDA formation (Ferysiuk & Wójciak, 2020). Lipid oxidation in muscle foods is strongly influenced by heme proteins and heme-derived iron pathways, whereas nitrite-derived nitric oxide can bind to and stabilize heme iron, thereby limiting the release of pro-oxidant free iron (Wu et al., 2024; Yong et al., 2021). Unlike sodium nitrite, which supplies a defined curing compound, the whole fermented preparations simultaneously introduced pre-converted nitrite, plant solids, and multiple plant-derived compounds into the sausage matrix. In muscle foods, the effectiveness of phenolic compounds depends on their localization, partitioning into lipid membranes, and interactions with heme proteins. Their antioxidant or pro-oxidant behavior may also vary with concentration, pH, and the presence of transition metals (Wu et al., 2024). Therefore, the consistently higher TBARS values in R6 and B6 than in the corresponding 3% treatments may reflect concentration- and matrix-dependent effects of the whole fermented vegetable systems, in which the accessibility of plant-derived compounds to lipid- and heme-oxidation sites may have differed between the two addition levels.

Overall, PC provided the most consistent lipid oxidation control during refrigerated storage. Among the natural treatments, R3 showed the best response at day 5, with low TBARS (0.39 mg/kg) together with the highest DPPH inhibition reported in the bioactive section (75.17%), whereas B3 showed the best late-stage TBARS value at day 10 (0.35 mg/kg). In contrast, the higher TBARS values in R6 and B6 indicate that increasing the fermented vegetable powder to 6% did not improve lipid oxidation control under the present conditions. These support the need to evaluate natural nitrite alternatives using multiple indicators, including residual nitrite, lipid oxidation, phenolic content, DPPH response, color stability, total viable count, and sensory acceptability.

### 3.6 External and internal color attributes

Table 5 shows that exterior and interior color parameters were significantly affected by treatment, storage time, and their interaction. The main treatment effect was observed in lightness and yellowness, where beetroot treatments, especially B6, produced a darker and more yellow color profile. For exterior L*, B6 showed the lowest values throughout storage, 30.72 at day 0, 28.89 at day 5, and 33.90 at day 10, while B3 was also darker than NC, PC, and radish treatments. A similar trend was observed internally, where B6 remained the darkest treatment from day 0 to day 10. This agrees with the pigment contribution of beetroot, which contains betalain pigments, including red-violet betacyanins and yellow betaxanthins, and has been reported to affect sausage color depending on the amount used (Sucu & Turp, 2018). In fermented dry sausages, Ozaki et al. (2021) also reported that beetroot powder affected L* and other color parameters during processing and storage, with the effect linked to betalain pigments.

**Table 5:** Exterior and interior color properties of beef sausages treated with fermented radish and beetroot powders during refrigerated storage.

Parameter		Storage	Treatments						Pₜ	Pₛ	Pₜ × ₛ
			NC	PC	R3	R6	B3	B6			
Exterior	L*	0	41.19 ± 0.63^abB^	41.89 ± 0.89^abA^	43.11 ± 1.14^aA^	40.72 ± 1.68^bA^	36.21 ± 1.33^cA^	30.72 ± 0.86^dB^	⁎⁎⁎	⁎	⁎⁎⁎
		5	44.21 ± 0.64^aA^	44.02 ± 1.62^aA^	41.95 ± 1.28^aA^	42.76 ± 1.22^aA^	32.26 ± 2.63^bB^	28.89 ± 2.43^cB^			
		10	45.14 ± 0.88^aA^	43.40 ± 2.28^abA^	39.81 ± 0.21^cB^	41.92 ± 1.37^bcA^	37.33 ± 1.11^dA^	33.90 ± 0.35^eA^			
	a*	0	11.93 ± 0.37^bA^	13.20 ± 0.81^aA^	7.40 ± 1.09^cA^	6.86 ± 0.26^cA^	6.89 ± 0.54^cA^	6.93 ± 0.19^cA^	⁎⁎⁎	⁎⁎⁎	⁎⁎⁎
		5	10.49 ± 0.16^aB^	9.25 ± 0.60^bB^	5.59 ± 0.84^dB^	4.15 ± 0.48^eB^	6.90 ± 0.72^cA^	6.04 ± 0.60^cdB^			
		10	3.46 ± 0.38^dC^	12.47 ± 0.88^aA^	4.95 ± 0.27^bB^	3.76 ± 0.56^cdB^	3.58 ± 0.45^dB^	4.63 ± 0.40^bcC^			
	b*	0	7.06 ± 0.22^deB^	6.64 ± 0.36^eAB^	8.12 ± 0.05^cdA^	9.57 ± 0.46^abA^	8.78 ± 0.57^bcB^	10.05 ± 1.29^aB^	⁎⁎⁎	⁎⁎⁎	⁎⁎⁎
		5	8.41 ± 0.55^cA^	6.43 ± 0.22^dB^	8.62 ± 0.34^cA^	10.57 ± 1.48^bA^	11.37 ± 0.92^abA^	12.41 ± 1.22^aA^			
		10	9.05 ± 0.38^bA^	7.07 ± 0.26^cA^	8.60 ± 0.30^bA^	8.65 ± 0.83^bA^	8.60 ± 0.40^bB^	11.30 ± 0.36^aAB^			

Interior	L*	0	44.75 ± 1.45^aB^	39.66 ± 0.76^cA^	41.49 ± 0.93^bA^	39.95 ± 0.11^cA^	31.72 ± 0.59^dC^	29.77 ± 0.28^eC^	⁎⁎⁎	⁎⁎⁎	⁎⁎⁎
		5	43.36 ± 0.56^aB^	42.06 ± 0.85^bA^	39.82 ± 0.29^cB^	39.84 ± 0.60^cA^	34.17 ± 0.64^dB^	31.79 ± 0.51^eB^			
		10	48.48 ± 0.30^aA^	41.58 ± 1.84^bA^	39.14 ± 0.44^cB^	38.62 ± 0.15^cB^	39.23 ± 1.39^cA^	32.86 ± 0.51^dA^			
	a*	0	16.08 ± 1.39^aA^	13.94 ± 0.31^bB^	9.36 ± 0.31^dB^	8.41 ± 0.03^deA^	10.66 ± 0.70^cA^	7.77 ± 0.19^eB^	⁎⁎⁎	⁎⁎⁎	⁎⁎⁎
		5	15.05 ± 0.26^aA^	10.51 ± 0.55^bC^	8.92 ± 0.24^dB^	7.18 ± 0.09^eB^	9.57 ± 0.34^cB^	6.41 ± 0.10^fC^			
		10	15.44 ± 1.89^bA^	17.29 ± 0.21^aA^	10.32 ± 0.33^cA^	6.77 ± 0.19^eC^	10.26 ± 0.09^cAB^	8.74 ± 0.16^dA^			
	b*	0	9.89 ± 0.93^cA^	9.66 ± 0.11^cA^	11.99 ± 1.04^bA^	12.73 ± 0.27^bA^	12.98 ± 0.38^bA^	14.59 ± 0.25^aA^	⁎⁎⁎	⁎⁎⁎	⁎⁎⁎
		5	9.01 ± 0.25^eA^	7.99 ± 0.07^fB^	11.90 ± 0.13^dAB^	12.90 ± 0.44^cA^	13.41 ± 0.12^bA^	15.06 ± 0.35^aA^			
		10	6.90 ± 0.32^fB^	9.61 ± 0.09^eA^	10.71 ± 0.21^dB^	12.29 ± 0.22^cA^	13.42 ± 0.11^bA^	14.82 ± 0.05^aA^			

Data is presented as mean ± SD. Within each storage day, different lowercase superscripts indicate significant differences among formulations; within each formulation, different uppercase superscripts indicate significant differences among storage days (p < 0.05). NC, negative control; PC, positive control; R3 and R6, fermented radish at 3% and 6%; B3 and B6, fermented beetroot at 3% and 6%. Pₜ, treatment effect; Pₛ, storage effect; Pₜ × ₛ, treatment × storage interaction.

⁎ P < 0.05.

⁎⁎⁎ P < 0.001.

Redness showed a different pattern between exterior and interior surfaces. For exterior color, PC showed the highest a* at day 0 and day 10, 13.20 and 12.47, respectively, indicating better maintenance of the typical nitrite-cured surface redness by the end of storage. This agrees with the role of nitrite in cured color formation, where nitrite-derived nitric oxide reacts with ferrous myoglobin to form nitrosylmyoglobin, which contributes to the characteristic cured-red color of meat products (Yong et al., 2021). Among the natural treatments, redness development was source- and position-dependent. B3 showed the highest exterior a* among the natural treatments at day 5 (6.90), whereas R3 showed the highest natural-treatment value at day 10 (4.95). For interior color, B3 maintained the highest a* among the natural treatments at day 0 and day 5, 10.66 and 9.57, respectively, while R3 and B3 were similar at day 10, 10.32 and 10.26. These results suggest that the fermented vegetable matrices contributed to redness retention in some cases, particularly B3 for the interior surface, but did not fully reproduce the cured-red color of PC, especially at day 10 when PC reached 12.47 for exterior a* and 17.29 for interior a*. Similar studies on pre-converted vegetable nitrite sources reported that natural nitrate/nitrite systems can support cured color development, although their effectiveness depends on the vegetable source, nitrite conversion, pigment composition, and meat matrix (Choi et al., 2017; Hwang et al., 2018). At day 5, exterior a* was highest in NC (10.49), but this should not be interpreted as cured color stability. Because NC did not contain nitrite or fermented vegetable matrix, its redness was more likely associated with native beef myoglobin forms and the absence of pigment from plant materials. This interpretation is supported by the sharp decline in exterior a* of NC by day 10 (3.46), whereas PC maintained a much higher value (12.47). Therefore, the high redness of NC at day 5 reflected a temporary native pigment response, not stable cured color development. Myoglobin is the main pigment responsible for meat color, while nitrite-derived nitric oxide participates in nitrosylmyoglobin formation in cured systems (Yong et al., 2021).

The yellowness results confirm that beetroot changed the color character. Exterior b* was highest in B6 across storage, 10.05, 12.41, and 11.30, and interior b* was also consistently highest in B6, reaching 14.82 at day 10. This shift is consistent with the contribution of beetroot betalain pigments, including betaxanthins, and with reports that the color characteristics of naturally cured products depend strongly on the plant source used (Ozaki et al., 2021; Sucu & Turp, 2018). Therefore, the beetroot treatments, particularly B6, produced a darker and more yellow color profile that may be visually different from the conventional cured color of PC.

The contribution of beetroot pigments should be distinguished from nitrite-mediated cured pigment formation. Beetroot-derived pigments can directly alter instrumental L*, a*, and b* values, whereas conventional cured redness in raw meat results mainly from the binding of nitrite-derived nitric oxide to ferrous myoglobin and the subsequent formation of nitrosylmyoglobin (Yong et al., 2021). In the present study, B6 consistently showed the lowest L* and highest b* values, while PC maintained higher a* values at day 0 and day 10. This pattern is more consistent with a substantial direct contribution from beetroot pigments than with greater cured-pigment formation in B6. Hwang et al. (2018) similarly reported that sausages containing pre-converted vegetable nitrite sources, including red beet, generally showed lower redness and higher yellowness than the sodium nitrite control. Because a colorimeter records the combined optical response of all pigments but does not chemically identify the responsible pigment, beetroot-derived color cannot be separated from nitrosylmyoglobin using L*, a*, and b* values alone.

Overall, PC remained the most effective treatment for maintaining the typical cured-red appearance, especially at day 10. Natural treatments modified the color in a source and level-dependent manner. B3 was the most promising natural treatment for internal redness, whereas B6 produced the strongest darkening and yellowness. These results suggest that the fermented vegetable matrices contributed to color development, but their effect was not equivalent to that of sodium nitrite and was influenced by plant pigments and nitrite availability.

### 3.7 Microbiological load and pH

Table 6 shows that total viable count and pH were significantly affected by treatment, storage time, and their interaction, indicating that microbial load and acidity changed depending on the treatment manner during refrigerated storage. At day 0, microbial counts ranged from 5.08 to 5.66 log₁₀ CFU/g, with B3 showing the lowest value and NC the highest. The main treatment separation appeared at the practical storage point, day 5, when NC increased markedly to 7.06 log₁₀ CFU/g, whereas all treated samples remained lower. Among them, R6 showed the lowest count (4.86 log₁₀ CFU/g). By day 10, the counts declined in all treatments, with B3 and R3 showing the lowest values, 4.02 and 4.21 log₁₀ CFU/g, respectively, both lower than PC (4.77 log₁₀ CFU/g) and NC (5.05 log₁₀ CFU/g). These results suggest that some fermented vegetable treatments, particularly R6 at day 5 and B3 and R3 at day 10, were associated with improved microbial quality compared with NC during refrigerated storage.

**Table 6:** Microbiological load and pH of beef sausage treated with fermented radish and beetroot powders during refrigerated storage.

Parameter	Storage	Treatments						Pₜ	Pₛ	Pₜ × ₛ
		NC	PC	R3	R6	B3	B6			
log₁₀ CFU/g	0	5.66 ± 0.07^aB^	5.60 ± 0.04^abA^	5.53 ± 0.06^abcA^	5.39 ± 0.08^cA^	5.08 ± 0.04^dB^	5.51 ± 0.14^bcB^	⁎⁎⁎	⁎⁎⁎	⁎⁎⁎
	5	7.06 ± 0.09^aA^	5.56 ± 0.12^cA^	5.21 ± 0.15^dB^	4.86 ± 0.03^eB^	5.73 ± 0.10^bcA^	5.88 ± 0.08^bA^			
	10	5.05 ± 0.04^aC^	4.77 ± 0.06^bB^	4.21 ± 0.04^dC^	4.81 ± 0.06^bB^	4.02 ± 0.16^eC^	4.52 ± 0.00^cC^			

pH	0	6.01 ± 0.04^aA^	5.94 ± 0.00^aA^	5.75 ± 0.02^bA^	5.58 ± 0.03^cA^	5.92 ± 0.01^aA^	5.76 ± 0.15^bA^	⁎⁎⁎	⁎⁎⁎	⁎⁎⁎
	5	5.68 ± 0.10^bB^	5.84 ± 0.00^aB^	5.52 ± 0.00^cB^	5.42 ± 0.04^dB^	5.76 ± 0.01^bB^	5.68 ± 0.01^bA^			
	10	5.42 ± 0.01^bC^	5.54 ± 0.03^aC^	5.40 ± 0.01^bC^	5.42 ± 0.05^bB^	5.19 ± 0.01^dC^	5.32 ± 0.01^cB^			

Data is presented as mean ± SD. Within each storage day, different lowercase superscripts indicate significant differences among formulations; within each formulation, different uppercase superscripts indicate significant differences among storage days (p < 0.05). NC, negative control; PC, positive control; R3 and R6, fermented radish at 3% and 6%; B3 and B6, fermented beetroot at 3% and 6%. Pₜ, treatment effect; Pₛ, storage effect; Pₜ × ₛ, treatment × storage interaction.

⁎⁎⁎ P < 0.001.

The lower microbial counts in several natural treatments may be related partly to acidification and to the presence of fermentation-derived inhibitory compounds in the whole fermented vegetable matrices. The pH decreased in all treatments during storage, from 6.01 to 5.42 in NC and from 5.94 to 5.54 in PC, while most natural treatments showed equal or lower final pH values. At day 5, R6 had the lowest pH (5.42), agreeing with the lowest microbial count (4.86 log₁₀ CFU/g). At day 10, B3 showed the lowest pH (5.19) and the lowest microbial count (4.02 log₁₀ CFU/g). This pattern suggests that acidification contributed to microbial control, although it was not the only factor, because microbial differences also depended on treatment source, concentration, and storage time. A comparable response was reported by Choi et al. (2017), where pre-converted red beet extract had a low pH, and the control showed the highest total viable count, while fermented red beet treatments, particularly when combined with ascorbic acid, showed lower TVC.

Nitrite-related effects may also have contributed to microbial control in PC and in the fermented vegetable treatments, but residual nitrite alone did not fully explain the microbial pattern. Nitrite is widely recognized for contributing to microbial control in processed meats, including inhibition of important foodborne and anaerobic bacteria (Yong et al., 2021). However, in the present study, the lowest microbial counts appeared in different natural treatments at different storage times, suggesting that microbial load was influenced by the combined effects of pH, residual nitrite, and fermentation-derived or plant-derived compounds in the whole fermented vegetable matrix.

Overall, R6 showed the strongest microbial control at day 5, while B3 and R3 showed the lowest counts at day 10. The pH results indicate that fermented radish and beetroot powders promoted a more acidified sausage matrix than PC in several cases, which may have supported microbial quality during storage. However, the response was treatment-dependent, suggesting that plant source and inclusion level should be optimized to balance microbial control with other quality attributes such as color, lipid oxidation, texture, and sensory acceptability.

### 3.8 External and internal texture profile

Table 7 shows that the TPA response was attribute-dependent. In both exterior and interior measurements, hardness and chewiness were the clearest texture indicators, as both were significantly affected by treatment, storage time, and their interaction. In contrast, cohesiveness, springiness, and adhesion showed smaller or less consistent responses, indicating that the main textural differences among treatments were related mainly to firmness and chewing resistance.

**Table 7:** Texture analysis profile (TPA) of beef sausage treated with fermented radish and beetroot powders during refrigerated storage.

Parameter		Storage	Treatments						P_t_	P_s_	P_txs_
			NC	PC	R3	R6	B3	B6			
Exterior	Hardness (kg)	0	0.04 ± 0.00^cA^	0.03 ± 0.00^dC^	0.04 ± 0.00^cB^	0.06 ± 0.00^aB^	0.05 ± 0.00^bB^	0.05 ± 0.00^bB^	⁎⁎⁎	⁎⁎⁎	⁎⁎⁎
		5	0.04 ± 0.00^dB^	0.04 ± 0.00^dB^	0.07 ± 0.00^aA^	0.06 ± 0.01^bB^	0.05 ± 0.00^cB^	0.06 ± 0.00^bB^			
		10	0.03 ± 0.00^dB^	0.05 ± 0.00^cA^	0.07 ± 0.01^bA^	0.08 ± 0.00^aA^	0.07 ± 0.00^bA^	0.08 ± 0.01^abA^			
	Cohesiveness	0	0.93 ± 0.02	0.93 ± 0.01	0.95 ± 0.00	0.95 ± 0.02	0.95 ± 0.01	0.94 ± 0.02	ns	⁎⁎⁎	ns
		5	0.92 ± 0.02	0.90 ± 0.03	0.90 ± 0.05	0.87 ± 0.05	0.91 ± 0.02	0.91 ± 0.02			
		10	0.94 ± 0.02	0.92 ± 0.02	0.93 ± 0.01	0.91 ± 0.00	0.91 ± 0.01	0.90 ± 0.02			
	Springiness (mm)	0	0.40 ± 0.00	0.50 ± 0.00	0.50 ± 0.00	0.50 ± 0.00	0.50 ± 0.00	0.50 ± 0.00	⁎⁎⁎	⁎⁎	ns
		5	0.43 ± 0.06	0.43 ± 0.06	0.43 ± 0.06	0.43 ± 0.06	0.50 ± 0.00	0.50 ± 0.00			
		10	0.47 ± 0.06	0.50 ± 0.00	0.50 ± 0.00	0.50 ± 0.00	0.50 ± 0.00	0.50 ± 0.00			
	Adhesion (mJ)	0	0.02 ± 0.01^aA^	0.02 ± 0.01^aA^	0.01 ± 0.01^aA^	0.01 ± 0.01^aB^	0.01 ± 0.01^aA^	0.01 ± 0.01^aA^	⁎	ns	⁎
		5	0.10 ± 0.10^aA^	0.01 ± 0.01^bA^	0.01 ± 0.01^bA^	0.02 ± 0.01^bA^	0.01 ± 0.01^bA^	0.01 ± 0.01^bA^			
		10	0.01 ± 0.01^aA^	0.01 ± 0.01^aA^	0.01 ± 0.01^aA^	0.01 ± 0.01^aAB^	0.01 ± 0.01^aA^	0.02 ± 0.01^aA^			
	Chewiness (kg*mm)	0	0.02 ± 0.00^dA^	0.01 ± 0.00^dB^	0.02 ± 0.00^cB^	0.03 ± 0.00^aB^	0.02 ± 0.00^bcB^	0.02 ± 0.00^bB^	⁎⁎⁎	⁎⁎⁎	⁎⁎⁎
		5	0.01 ± 0.00^bA^	0.01 ± 0.00^bB^	0.03 ± 0.01^aA^	0.02 ± 0.01^aB^	0.02 ± 0.00^abB^	0.03 ± 0.00^aB^			
		10	0.01 ± 0.00^dA^	0.02 ± 0.00^cA^	0.03 ± 0.00^abA^	0.04 ± 0.00^aA^	0.03 ± 0.00^bA^	0.03 ± 0.00^abA^			

Interior	Hardness (kg)	0	0.02 ± 0.00^cC^	0.03 ± 0.00^bB^	0.03 ± 0.00^bC^	0.04 ± 0.00^aA^	0.04 ± 0.00^aB^	0.04 ± 0.00^aB^	⁎⁎⁎	⁎⁎⁎	⁎⁎⁎
		5	0.04 ± 0.00^bA^	0.03 ± 0.00^cB^	0.07 ± 0.00^aA^	0.04 ± 0.01^bA^	0.07 ± 0.00^aA^	0.06 ± 0.00^aA^			
		10	0.03 ± 0.00^cB^	0.05 ± 0.01^bA^	0.05 ± 0.00^bB^	0.05 ± 0.01^bA^	0.07 ± 0.00^aA^	0.04 ± 0.00^cC^			
	Cohesiveness	0	1.05 ± 0.14	0.93 ± 0.03	0.88 ± 0.03	0.91 ± 0.05	0.92 ± 0.05	0.93 ± 0.03	ns	ns	ns
		5	0.93 ± 0.05	0.93 ± 0.05	0.92 ± 0.04	0.90 ± 0.05	0.91 ± 0.03	0.93 ± 0.04			
		10	0.92 ± 0.03	0.92 ± 0.02	0.90 ± 0.03	0.91 ± 0.03	0.94 ± 0.02	0.89 ± 0.04			
	Springiness (mm)	0	0.50 ± 0.17	0.47 ± 0.06	0.43 ± 0.06	0.47 ± 0.06	0.47 ± 0.06	0.47 ± 0.06	ns	ns	ns
		5	0.47 ± 0.06	0.43 ± 0.06	0.47 ± 0.06	0.47 ± 0.06	0.47 ± 0.06	0.47 ± 0.06			
		10	0.43 ± 0.06	0.47 ± 0.06	0.43 ± 0.06	0.43 ± 0.06	0.50 ± 0.00	0.43 ± 0.06			
	Adhesion (mJ)	0	0.05 ± 0.02	0.06 ± 0.01	0.02 ± 0.01	0.03 ± 0.01	0.04 ± 0.01	0.02 ± 0.00	⁎⁎⁎	ns	ns
		5	0.06 ± 0.01	0.04 ± 0.01	0.02 ± 0.01	0.02 ± 0.01	0.03 ± 0.00	0.02 ± 0.01			
		10	0.04 ± 0.02	0.05 ± 0.02	0.02 ± 0.02	0.01 ± 0.01	0.04 ± 0.01	0.03 ± 0.01			
	Chewiness (kg*mm)	0	0.01 ± 0.01^aA^	0.01 ± 0.00^aAB^	0.01 ± 0.00^aB^	0.02 ± 0.00^aA^	0.02 ± 0.00^aB^	0.02 ± 0.00^aAB^	⁎⁎⁎	⁎⁎⁎	⁎⁎⁎
		5	0.02 ± 0.00^bA^	0.01 ± 0.00^bB^	0.03 ± 0.01^aA^	0.02 ± 0.01^bA^	0.03 ± 0.01^aA^	0.03 ± 0.01^aA^			
		10	0.01 ± 0.00^bA^	0.02 ± 0.00^bA^	0.02 ± 0.00^bB^	0.02 ± 0.01^bA^	0.03 ± 0.00^aA^	0.01 ± 0.00^bB^			

Data is presented as mean ± SD. Within each storage day, different lowercase superscripts indicate significant differences among formulations; within each formulation, different uppercase superscripts indicate significant differences among storage days (p < 0.05). NC, negative control; PC, positive control; R3 and R6, fermented radish at 3% and 6%; B3 and B6, fermented beetroot at 3% and 6%, Pt; treatment, Ps; storage, Ptxs; treatment vs storage. NS-not significant.

⁎ P < 0.05.

⁎⁎ P < 0.01.

⁎⁎⁎ P < 0.001.

For exterior texture, the natural treatments generally produced firmer samples than PC and NC during storage. At the practical storage point, day 5, R3 showed the highest exterior hardness (0.07 kg), followed by R6 and B6 (0.06 kg), whereas NC and PC were lower (0.04 kg). By day 10, R6 and B6 reached the highest exterior hardness values (0.08 kg), while PC remained lower (0.05 kg) and NC was the softest (0.03 kg). Exterior chewiness followed a similar pattern, with R3 and B6 showing the highest values at day 5 (0.03 kg*mm), and R6 recording the highest value at day 10 (0.04 kg*mm). This firmer texture may be related to the incorporation of whole fermented vegetable matrices, which increased plant-derived solids and could have modified water binding and the protein-fiber structure of the sausage matrix. Similar effects have been reported in meat products formulated with plant-based powders or hydrocolloid-type ingredients, where these materials improved water and fat retention and modified matrix structure through swelling, binding, and water-entrapment effects (Hawashin et al., 2016; Kim et al., 2025).

For interior texture, hardness, and chewiness again showed the strongest treatment separation. At day 5, R3, B3, and B6 showed the highest interior hardness values (0.07, 0.07, and 0.06 kg, respectively), all higher than PC (0.03 kg) and NC (0.04 kg). By day 10, B3 remained the firmest treatment (0.07 kg), while PC, R3, and R6 were intermediate (0.05 kg), and NC and B6 were lower. Interior chewiness was also highest in R3, B3, and B6 at day 5 (0.03 kg*mm), and B3 retained the highest value at day 10. The higher interior hardness and chewiness in R3, B3, and B6 suggest that the whole fermented vegetable matrices modified the internal sausage structure. This may be related to the addition of plant-derived solids and fiber material, which can increase water immobilization, improve emulsion stability, and support structural integrity in processed meat systems (Kim et al., 2025).

Overall, fermented radish and beetroot powders modified the sausage texture did not produce the same soft texture profile observed in PC. R3, R6, and B3 contributed most clearly to firmness and chewiness. The texture data suggest that fermented vegetable matrices can increase structural firmness, but the inclusion level and plant source should be optimized to avoid excessive chewiness while maintaining sensory acceptability.

### 3.9 Sensory analysis

Table 8 shows that the sensory response differed among sensory attributes. Color, texture, tenderness, and overall acceptability were significantly affected by treatment, storage time, and their interaction, indicating that panel perception changed according to both formulation and storage stage. In contrast, taste was affected only by storage time, while flavor was significantly affected by treatment and storage time without a significant interaction. This showed that taste deterioration was mainly storage-related, whereas color, texture, tenderness, and overall acceptability were more sensitive to the specific treatment used.

**Table 8:** Sensory analysis of beef sausage treated with fermented radish and beetroot powders during refrigerated storage.

Parameter	Storage	Treatments						P_t_	P_s_	P_txs_
		NC	PC	R3	R6	B3	B6			
Color	0	5.58 ± 1.31^bA^	7.25 ± 1.66^aA^	5.50 ± 2.24^bA^	4.83 ± 2.08^bA^	5.00 ± 0.95^bA^	4.08 ± 1.51^bB^	⁎⁎	⁎⁎	⁎⁎
	5	5.58 ± 1.24^abA^	6.83 ± 1.99^aA^	4.00 ± 1.41^bB^	4.33 ± 1.56^bA^	4.17 ± 1.19^bA^	4.42 ± 0.67^bAB^			
	10	3.67 ± 0.98^bB^	6.42 ± 2.27^aA^	4.00 ± 1.21^bB^	4.33 ± 1.15^bA^	4.75 ± 0.97^bA^	5.17 ± 1.11^abA^			

Taste	0	6.42 ± 1.51	6.75 ± 1.36	6.67 ± 1.87	6.67 ± 2.27	5.75 ± 2.34	6.42 ± 2.11	ns	⁎⁎	ns
	5	4.75 ± 1.96	4.58 ± 2.31	4.42 ± 2.91	4.58 ± 1.88	4.42 ± 2.47	4.83 ± 1.27			
	10	3.50 ± 1.09	5.08 ± 2.19	5.50 ± 2.28	5.08 ± 1.78	4.58 ± 1.56	5.17 ± 2.21			

Texture	0	5.17 ± 0.94^bA^	7.58 ± 0.90^aA^	4.58 ± 1.44^bA^	5.83 ± 1.90^bA^	5.58 ± 1.88^bA^	5.75 ± 1.06^bA^	⁎⁎	⁎⁎	⁎⁎
	5	3.83 ± 1.59^aB^	4.33 ± 1.50^aB^	4.75 ± 2.09^aA^	4.92 ± 1.93^aAB^	5.00 ± 1.91^aAB^	4.00 ± 1.13^aB^			
	10	3.00 ± 0.85^bB^	4.83 ± 2.17^aB^	4.33 ± 0.78^abA^	4.50 ± 0.80^aB^	4.00 ± 1.04^abB^	4.83 ± 1.70^aAB^			

Flavor	0	5.67 ± 1.15	6.75 ± 1.76	6.33 ± 1.78	6.83 ± 1.64	6.25 ± 1.86	6.00 ± 1.86	⁎	⁎⁎	ns
	5	4.17 ± 1.64	4.17 ± 1.27	4.42 ± 3.06	4.17 ± 2.17	4.50 ± 2.02	4.33 ± 1.44			
	10	2.67 ± 0.78	4.75 ± 2.01	3.83 ± 2.29	3.83 ± 1.11	3.75 ± 1.60	4.58 ± 2.19			

Tenderness	0	6.08 ± 1.68^abA^	6.67 ± 0.98^aA^	4.75 ± 2.01^bA^	5.50 ± 2.11^abA^	5.08 ± 1.98^abA^	5.33 ± 1.61^abA^	⁎⁎	⁎⁎	⁎⁎
	5	2.67 ± 1.15^bB^	3.42 ± 1.00^abB^	4.50 ± 2.07^aA^	3.75 ± 2.42^abB^	4.67 ± 1.83^aA^	5.08 ± 1.44^aA^			
	10	2.50 ± 0.67^bB^	4.67 ± 2.35^aB^	2.67 ± 0.65^bB^	3.67 ± 1.23^abB^	3.58 ± 1.08^abA^	4.17 ± 1.64^abA^			

Overall Acceptability	0	5.92 ± 0.67^abA^	7.25 ± 0.97^aA^	6.08 ± 1.51^abA^	6.25 ± 1.96^abA^	5.67 ± 1.72^bA^	5.83 ± 1.53^abA^	⁎⁎	⁎⁎	⁎
	5	4.08 ± 1.51^aB^	4.25 ± 1.29^aB^	4.58 ± 2.39^aB^	4.33 ± 2.06^aB^	4.50 ± 2.11^aAB^	4.92 ± 1.56^aA^			
	10	3.08 ± 1.00^bB^	5.25 ± 2.34^aB^	4.25 ± 1.54^abB^	4.25 ± 1.54^abB^	4.33 ± 1.23^abB^	5.42 ± 2.31^aA^			

Data is presented as mean ± SD. Within each storage day, different lowercase superscripts indicate significant differences among formulations; within each formulation, different uppercase superscripts indicate significant differences among storage days (p < 0.05). NC, negative control; PC, positive control; R3 and R6, fermented radish at 3% and 6%; B3 and B6, fermented beetroot at 3% and 6%, Pt; treatment, Ps; storage, Ptxs; treatment vs storage. NS-not significant.

⁎ P < 0.05.

⁎⁎ P < 0.001.

Sensory color clearly favored PC during the early and practical storage periods. PC received the highest color scores at day 0 and day 5, with values of 7.25 and 6.83, respectively, confirming the positive contribution of conventional nitrite curing to visual acceptability. This agrees with the known importance of cured color in processed meats, where nitrite-derived pigment formation supports the characteristic cured appearance that strongly affects consumer perception (Melios et al., 2024). Choi et al. (2017) similarly reported that meat emulsions containing nitrite and ascorbic acid had the highest color score. By day 10, PC still had the highest numerical color score (6.42), but B6 increased to 5.17 and became statistically comparable to PC, while NC decreased to 3.67. However, this sensory similarity did not mean that B6 had the same instrumental color profile as PC. At day 10, B6 was darker and more yellow than PC, with lower exterior L* (33.90 versus 43.40), lower exterior a* (4.63 versus 12.47), and higher exterior b* (11.30 versus 7.07). Internally, B6 also showed lower L* (32.86 versus 41.58), lower a* (8.74 versus 17.29), and higher b* (14.82 versus 9.61) than PC. Therefore, the comparable sensory color scores of B6 and PC at day 10 suggest that panelists accepted a darker, more yellow beetroot-derived appearance, although this profile was instrumentally different from the typical nitrite-cured color of PC. This interpretation is consistent with Choi et al. (2017), who reported that a meat emulsion containing 10% pre-converted red beet extract with ascorbic acid had overall acceptability comparable to the nitrite and ascorbic acid treatment.

Taste was affected by storage time, but not by treatment or the treatment x storage interaction. At day 0, taste scores were generally high across treatments, ranging from 5.75 in B3 to 6.75 in PC. At day 5, the scores decreased in all treatments, ranging from 4.42 to 4.83, indicating that the practical storage point was associated with a general decline in taste perception. At day 10, several treatments showed partial recovery or maintenance, with R3, B6, PC, and R6 recording 5.50, 5.17, 5.08, and 5.08, respectively, while NC remained the lowest at 3.50. However, because treatment and interaction effects were not significant for taste, these numerical differences should be discussed cautiously as descriptive trends only.

Flavor was significantly affected by treatment and storage time, whereas the treatment x storage interaction was not significant, indicating that flavor differences among treatments were relatively consistent across storage. Flavor scores declined from day 0 to day 5 in all treatments, suggesting that the practical storage point was associated with a general reduction in flavor perception. This storage-related decline agrees with Hwang et al. (2017), who reported that flavor scores in low-salt frankfurters containing fermented red beet decreased as refrigerated storage progressed, indicating that sensory quality can decline during storage even when fermented vegetable curing ingredients are used. At day 10, NC showed the lowest numerical flavor score (2.67), while PC and B6 showed the highest numerical values (4.75 and 4.58, respectively).

Textures as sensory attributes showed a treatment-dependent storage response. At day 0, PC received the highest texture and tenderness scores, 7.58 and 6.67, respectively, which agrees with its softer instrumental texture profile. This showed that the initial texture of PC was clearly preferred by panelists. At day 5, however, the natural treatments became more competitive. B6 showed the highest tenderness score (5.08), followed by B3 (4.67) and R3 (4.50), while NC and PC showed lower values, 2.67 and 3.42, respectively. This showed that, at the practical storage point, some fermented vegetable treatments maintained a more acceptable bite and tenderness than NC and were numerically higher than PC. By day 10, PC and B6 showed the highest sensory texture values, both at 4.83, while B6 also maintained a relatively high tenderness score (4.17). This response should not be interpreted only through instrumental hardness. For example, B6 at day 10 showed high exterior hardness, but lower interior hardness and chewiness than B3, suggesting that panelists may have perceived a firmer surface together with a less resistant internal bite. Therefore, sensory texture was influenced by the combined perception of surface firmness, internal bite, tenderness, and eating quality, not by hardness alone.

The overall acceptability followed the combined effects of color, texture, flavor, and tenderness. At day 0, PC had the highest overall acceptability score (7.25), confirming its early advantage as the conventional nitrite control. At day 5, differences among treatments became less clear, and B6 had the highest numerical overall acceptability score (4.92), followed by R3 (4.58), B3 (4.50), R6 (4.33), PC (4.25), and NC (4.08). By day 10, B6 reached the highest overall acceptability among all treatments (5.42) and was statistically comparable to PC (5.25), whereas NC showed the lowest value (3.08). This showed that B6 was the strongest natural treatment from a sensory perspective at the extended storage point. The result is consistent with reports that beetroot-based natural curing systems can maintain acceptable sensory quality in sausage-type products, although the response depends on the amount added and the product matrix. Sucu and Turp (2018) reported that sensory scores of fermented beef sausages containing beetroot powder were generally comparable to the nitrite control during storage, with some beetroot treatments showing similar or higher overall acceptability at certain storage points. Choi et al. (2017) also reported that meat emulsions containing 10% pre-converted red beet extract with ascorbic acid achieved overall acceptability comparable to the nitrite and ascorbic acid treatment.

Overall, the sensory results indicate that PC provided the strongest initial sensory quality, particularly for color, texture, tenderness, and overall acceptability. However, B6 showed the best sensory score among the natural treatments by day 10, with color, texture, tenderness, flavor, and overall acceptability values close to PC in several cases. This showed that the 6% fermented beetroot powder improved late-stage sensory acceptability, probably through its contribution to visual appearance and eating quality, although its performance was not uniform across all quality indicators. Therefore, B6 was the most promising natural treatment from a sensory perspective at day 10, as it showed the highest overall acceptability and better scores for color, texture, flavor, and tenderness. However, this sensory advantage was not matched by lipid oxidation control, residual nitrite retention, DPPH activity, or instrumental cured redness. Thus, B6 may improve late-stage sensory acceptability, but its formulation still requires optimization to balance sensory quality with oxidative stability.

### 3.10 Limitations

This study has several limitations. Although nitrate and nitrite concentrations were determined before and after fermentation, nitrate decrease (%) represents the relative decrease in measured nitrate concentration and does not quantify the nitrate-to-nitrite conversion rate. Furthermore, N-nitrosamines were not analyzed. Consequently, the proposed molecular mechanisms remain literature-supported interpretations, and lower residual nitrite cannot be considered direct evidence of reduced nitrosamine formation or improved product safety.

## 4 Conclusion

This study evaluated fermented radish and beetroot powders as pre-converted nitrite vegetables for their potential to improve beef sausage quality during refrigerated storage. Compared with NC, selected natural treatments improved specific quality responses, including bioactive properties, lipid oxidation, microbial load, and sensory acceptability. Among the natural treatments, fermented radish showed the most promising overall performance. R6 had the highest residual nitrite and phenolic content among the natural treatments at day 10 and was associated with lower microbial counts at day 5, while R3 showed the highest DPPH activity, the lowest TBARS value at the practical storage point, day 5, and lower microbial counts at day 10. Although radish showed stronger responses, beetroot treatments also provided positive effects, particularly during extended storage. B3 showed lower TBARS and microbial load, while B6 had sensory acceptability comparable to PC at the end of storage. Overall, fermented radish and beetroot powders have the potential to be natural curing ingredients in beef sausages, especially as whole vegetable matrices providing converted nitrite and bioactive compounds. However, comparison with PC indicates that additional optimization is still needed to improve consistency of color, microbial quality, oxidative stability, and sensory acceptability before these ingredients can be considered practical alternatives to conventional sodium nitrite.

## Funding and acknowledgements

This research project is supported by a grant from King Abdelaziz City for Science and Technology (KACST), represented by the Science, Technology and Innovation Unit at 10.13039/501100002383King Saud University (STU-KSU), Riyadh, Saudi Arabia (Grant No. STU-63).

## CRediT authorship contribution statement

**Samar A. Almohaimeed:** Writing – original draft, Software, Methodology, Investigation, Funding acquisition, Formal analysis, Data curation, Conceptualization. **Fahad Y. AL Juhaimi:** Writing – review & editing, Writing – original draft, Visualization, Validation, Supervision, Resources, Project administration, Investigation, Conceptualization. **Elfadil E. Babiker:** Writing – review & editing, Visualization, Validation, Supervision, Resources. **Isam A. Mohamed Ahmed:** Writing – review & editing, Writing – original draft, Visualization, Validation, Supervision, Software, Resources, Project administration, Methodology, Investigation, Formal analysis, Data curation, Conceptualization.

## Ethics declaration

Written informed consent to take part in the study and to publish the article has been obtained from all participants or their legal representatives. The privacy rights of participants have been observed.

This study was performed in compliance with relevant laws, regulatory frameworks and guidelines where the research took place.

This study was approved by the King Saud University Medical City, Institutional Review Board.

(Approval No. E-25-10195)

## Declaration of competing interest

The authors declare that they have no known competing financial interests or personal relationships that could have appeared to influence the work reported in this paper.

## Data Availability

Data will be made available on request.

## References

[bb0005] Al-Juhaimi F.Y., Mohamed Ahmed I.A., Adiamo O.Q., Adisa A.R., Gahfoor K., Özcan M.M., Babiker E.E. (2018). Effect of argel (*Solenostemma argel*) leaf powder on the quality attributes of camel patties during cold storage. Journal of Food Processing and Preservation.

[bb0010] Almohaimeed S.A., Al Juhaimi F.Y., Babiker E.E., Mohamed Ahmed I.A. (2026). Fermented radish and beetroot powder as natural sources of nitrite in beef burger. Foods.

[bb0015] AOAC (2007).

[bb0020] Bae S.M., Choi J.H., Jeong J.Y. (2020). Effects of radish powder concentration and incubation time on the physicochemical characteristics of alternatively cured pork products. Journal of Animal Science and Technology.

[bb0025] Bourne M.C. (1978). Texture profile analysis. Food Technology.

[bb0030] Chen L., Tang S., Li S., Li J., Li Q., Zhao J. (2023). Effect of malondialdehyde oxidation on the structure and functional properties of myofibrillar protein in yak meat. Food Science.

[bb0035] Choi Y.S., Kim T.K., Jeon K.H., Park J.D., Kim H.W., Hwang K.E., Kim Y.B. (2017). Effects of pre-converted nitrite from red beet and ascorbic acid on quality characteristics in meat emulsions. Food Science of Animal Resources.

[bb0040] Echegaray N., Pateiro M., Munekata P.E.S., Lorenzo J.M., Chabani Z., Farag M.A., Domínguez R. (2021). Measurement of antioxidant capacity of meat and meat products: Methods and applications. Molecules.

[bb0045] Ferysiuk, K., & Wójciak, K. M. (2020). Reduction of nitrite in meat products through the application of various plant-based ingredients. *Antioxidants 2020, vol. 9,* (8), *711*, doi:10.3390/ANTIOX9080711. PMC7464959 32764511

[bb0050] FSIS U. (1995).

[bb0055] Guimarães A.S., Guimarães J.S., Araújo A.B.S., Rodrigues L.M., Carvalho E.E.N., Ramos A.d.L.S., Ramos E.M. (2021). Characterization of natural curing agents from Japanese radish (*Raphanus sativus* L.) for their use in clean label restructured cooked meat products. LWT - Food Science and Technology.

[bb0060] Hawashin M.D., Al-Juhaimi F., Ahmed I.A.M., Ghafoor K., Babiker E.E. (2016). Physicochemical, microbiological and sensory evaluation of beef patties incorporated with destoned olive cake powder. Meat Science.

[bb0065] Hwang K.E., Kim T.K., Kim H.W., Oh N.S., Kim Y.B., Jeon K.H., Choi Y.S. (2017). Effect of fermented red beet extracts on the shelf stability of low-salt frankfurters. Food Science and Biotechnology.

[bb0070] Hwang K.E., Kim T.K., Kim H.W., Seo D.H., Kim Y.B., Jeon K.H., Choi Y.S. (2018). Effect of natural pre-converted nitrite sources on color development in raw and cooked pork sausage. Asian-Australasian Journal of Animal Sciences.

[bb0075] ICMSF, Clark D.S. (1983).

[bb0080] Jayawardana B.C., Hirano T., Han K.H., Ishii H., Okada T., Shibayama S., Shimada K.I. (2011). Utilization of adzuki bean extract as a natural antioxidant in cured and uncured cooked pork sausages. Meat Science.

[bb0085] Jeong J.Y., Bae S.M., Yoon J., Jeong D.H., Gwak S.H. (2020). Effect of using vegetable powders as nitrite/nitrate sources on the physicochemical characteristics of cooked pork products. Food Science of Animal Resources.

[bb0090] Kim M., Bae S.M., Yoo Y., Park J., Jeong J.Y. (2025). Clean-label strategies for the replacement of nitrite, ascorbate, and phosphate in meat products: A review. Foods.

[bb0095] Liu, R., Xing, L., Fu, Q., Zhou, G. H., & Zhang, W. G. (2016). A review of antioxidant peptides derived from meat muscle and by-products. *Antioxidants 2016, vol. 5, page 32*, *5*(3), 32. doi:10.3390/ANTIOX5030032. PMC5039581 27657142

[bb0100] McMullen S.E., Casanova J.A., Gross L.K., Schenck F.J. (2005). Ion chromatographic determination of nitrate and nitrite in vegetable and fruit baby foods. Journal of AOAC International.

[bb0105] Melios S., Grasso S., Bolton D., Crofton E. (2024). Sensory quality and consumer perception of reduced/free-from nitrates/nitrites cured meats. Current Opinion in Food Science.

[bb0110] Merino L. (2009). Development and validation of a method for determination of residual nitrite/nitrate in foodstuffs and water after zinc reduction. Food Analytical Methods.

[bb0115] Ozaki M.M., Munekata P.E.S., Jacinto-Valderrama R.A., Efraim P., Pateiro M., Lorenzo J.M., Pollonio M.A.R. (2021). Beetroot and radish powders as natural nitrite source for fermented dry sausages. Meat Science.

[bb0120] Ozdal T., Capanoglu E., Altay F. (2013). A review on protein–phenolic interactions and associated changes. Food Research International.

[bb0125] Özer C.O., Var G.B., Demir Özer E., Kiliç B. (2025). Effects of pomegranate flower extracts on antioxidant properties, phenolic content, and quality attributes of nitrite reduced chicken sausages. Animal Science Journal = Nihon Chikusan Gakkaiho.

[bb0130] Papastergiadis A., Mubiru E., Van Langenhove H., De Meulenaer B. (2012). Malondialdehyde measurement in oxidized foods: Evaluation of the spectrophotometric thiobarbituric acid reactive substances (TBARS) test in various foods. Journal of Agricultural and Food Chemistry.

[bb0135] Polizer-Rocha Y.J., Lorenzo J.M., Pompeu D., Rodrigues I., Baldin J.C., Pires M.A., Trindade M.A. (2020). Physicochemical and technological properties of beef burger as influenced by the addition of pea fibre. International Journal of Food Science and Technology.

[bb0140] Rosmini M.R., Perlo F., Pérez-Alvarez J.A., Pagán-Moreno M.J., Gago-Gago A., López-Santoveña F., Aranda-Catalá V. (1996). TBA test by an extractive method applied to ‘paté.’. Meat Science.

[bb0145] Schopfer B., Mitrenga S., Boulaaba A., Roolfs K., Plötz M., Becker A. (2022). Red beet and Swiss chard juice extract as natural nitrate sources for the production of alternatively-cured emulsion-type sausages. Meat Science.

[bb0150] Serdaroğlu M., Can H., Sarı B., Kavuşan H.S., Yılmaz F.M. (2023). Effects of natural nitrite sources from arugula and barberry extract on quality characteristic of heat-treated fermented sausages. Meat Science.

[bb0155] Siddiqui M.R., Wabaidur S.M., Khan M.A., Al Othman Z.A., Rafiquee M.Z.A., Alqadami A.A. (2018). A rapid and sensitive evaluation of nitrite content in Saudi Arabian processed meat and poultry using a novel ultra performance liquid chromatography–mass spectrometry method. Journal of Food Science and Technology.

[bb0160] Singh R.P., Chidambara Murthy K.N., Jayaprakasha G.K. (2002). Studies on the antioxidant activity of pomegranate (*Punica granatum*) peel and seed extracts using in vitro models. Journal of Agricultural and Food Chemistry.

[bb0165] Sucu C., Turp G.Y. (2018). The investigation of the use of beetroot powder in Turkish fermented beef sausage (sucuk) as nitrite alternative. Meat Science.

[bb0170] Tapia-Quirós, P., Mir-Cerdà, A., Granados, M., Sentellas, S., & Saurina, J. (2025). From waste to resource: Exploring green approaches for phenolics recovery from olive leaves. *Antioxidants 2025,* Vol 14*, 14*(2). doi:10.3390/antiox14020136. PMC11852040 40002323

[bb0175] Umaraw, P., Singh, V. P., & Verma, A. K. (2024). Effect of addition of mango seed extract on storage stability of Chevon meatballs at refrigeration temperature. *Foods 2024, vol. 13, page 676*, *13*(5), 676. doi:10.3390/FOODS13050676. PMC10930499 38472788

[bib191] Wang Y., Dominguez R., Lorenzo J.M., Bohrer B.M. (2021). The relationship between lipid content in ground beef patties with rate of discoloration and lipid oxidation during simulated retail display. Foods.

[bb0180] Wu H., Bak K.H., Goran G.V., Tatiyaborworntham N. (2024). Inhibitory mechanisms of polyphenols on heme protein-mediated lipid oxidation in muscle food: New insights and advances. Critical Reviews in Food Science and Nutrition.

[bb0185] Yong H.I., Kim T.K., Choi H.D., Jang H.W., Jung S., Choi Y.S. (2021). Clean label meat technology: Pre-converted nitrite as a natural curing. Food Science of Animal Resources.

[bb0190] Zhang Y., Zhang Y., Jia J., Peng H., Qian Q., Pan Z., Liu D. (2023). Nitrite and nitrate in meat processing: Functions and alternatives. Current Research in Food Science.

